# Dried blood spot specimen quality and validation of a new pre-analytical processing method for qualitative HIV-1 PCR, KwaZulu-Natal, South Africa

**DOI:** 10.4102/ajlm.v5i1.349

**Published:** 2016-02-25

**Authors:** Kerusha Govender, Raveen Parboosing, Ntombizandile Siyaca, Pravikrishnen Moodley

**Affiliations:** 1 Department of Virology, Inkosi Albert Luthuli Central Hospital, National Health Laboratory Service, Durban, KwaZulu-Natal, South Africa; 2 Department of Virology, School of Laboratory Medicine and Medical Sciences, University of KwaZulu-Natal, South Africa

## Abstract

**Background:**

Poor quality dried blood spot (DBS) specimens are usually rejected by virology laboratories, affecting early infant diagnosis of HIV. The practice of combining two incompletely-filled DBS in one specimen preparation tube during pre-analytical specimen processing (i.e., the two-spot method) has been implemented to reduce the number of specimens being rejected for insufficient volume.

**Objectives:**

This study analysed laboratory data to describe the quality of DBS specimens and the use of the two-spot method over a one-year period, then validated the two-spot method against the standard (one-spot) method.

**Methods:**

Data on HIV-1 PCR test requests submitted in 2014 to the Department of Virology at Inkosi Albert Luthuli Central Hospital in KwaZulu-Natal province, South Africa were analysed to describe reasons for specimen rejection, as well as results of the two-spot method. The accuracy, lower limit of detection and precision of the two-spot method were assessed.

**Results:**

Of the 88 481 specimens received, 3.7% were rejected for pre-analytical problems. Of those, 48.9% were rejected as a result of insufficient specimen volume. Two health facilities had significantly more specimen rejections than other facilities. The two-spot method prevented 10 504 specimen rejections. The Pearson correlation coefficient comparing the standard to the two-spot method was 0.997.

**Conclusions:**

The two-spot method was comparable with the standard method of pre-analytical specimen processing. Two health facilities were identified for targeted retraining on specimen quality. The two-spot method of DBS specimen processing can be used as an adjunct to retraining, to reduce the number of specimens rejected and improve linkage to care.

## Introduction

Early diagnosis and treatment of HIV-positive infants significantly reduce HIV disease progression and mortality. Early infant diagnosis of HIV-positive infants is a critical step in the timely initiation of antiretroviral therapy (ART).^1,2^ Despite these substantial benefits, achieving early infant diagnosis is challenging in resource-limited settings. Problems in the pre-analytical stages of specimen collection and processing lead to poor quality specimens and laboratory rejections.^3^ A meta-analysis of studies between 2001 and 2012 showed that 33.9% of HIV-exposed infants in sub-Saharan Africa are lost to follow up by three months of age.^4^ Early infant diagnosis in South Africa occurs within a Prevention of Mother-to-Child Transmission continuum of care. Qualitative HIV-1 DNA and RNA PCR testing is done on dried blood spot (DBS) specimens collected from HIV-exposed infants between six weeks and 18 months of age, using the COBAS^®^ AmpliPrep/COBAS^®^ TaqMan^®^ (CAP/CTM) HIV-1 Qualitative test (Roche^®^ Molecular Systems, Inc., Branchburg, New Jersey, United States). The current South African national laboratory guideline allows for testing of one completely-filled DBS per assay.^5^

There have been significant successes in the Prevention of Mother-to-Child Transmission programme. However, challenges have been identified in South Africa. Only 70.4% of HIV-exposed South African infants were tested for HIV by two months of age in 2011, reflecting a lack of implementation of policies in the field.^6^ The Department of Virology (DOV) in KwaZulu-Natal province in South Africa provides early infant diagnosis services for the province. Laboratory data from KwaZulu-Natal show a reduction in mother-to-child transmission of HIV which reflects national trends.^7^ However, there is a challenge of identifying and linking every HIV-exposed infant to care, which contributes to a significant number of HIV-positive infants not commencing ART in a timely manner.^8^

Laboratory specimen rejections are an essential part of a laboratory quality management system, allowing the technologist to optimise accuracy of results. However, this practice has also been shown to adversely impact patient care as it can lead to abandonment of the test request.^9,10^ Specimen rejection may play a role in the failure to link HIV-exposed infants to care. The challenges with DBS specimens in our setting have not been described.

In the standard method of pre-analytical specimen processing, one complete DBS is inserted into one specimen preparation tube. At the DOV, the current practice for DBS with blood spots that are smaller than the recommended size is to use two incomplete DBS and combine them in one specimen preparation tube during pre-analytical specimen processing (i.e., the two-spot method). Specimen preparation is followed by PCR testing for HIV-1 using the CAP/CTM analyser. The two-spot method is based on the hypothesis that the quality of these specimens may be adversely affected by the small volume of blood they contain. By maximising the amount of blood eluted from each specimen, the correct volume for the PCR can be obtained and a good quality laboratory result can be produced. Neither the two-spot method nor the impact of this method in reducing laboratory rejections have been described in the scientific literature.

The objectives of this study were to analyse DOV laboratory data to describe trends in DBS specimen quality, particularly relating to specimens with insufficient volume. In addition, we sought to describe the use of the two-spot method in routine HIV PCR diagnosis. Finally, we performed laboratory experiments to validate the two-spot method.

## Research method and design

### Ethical considerations

The University of KwaZulu-Natal Biomedical Research Ethics Committee (BREC) approved this research under the approval classes BCA-143/09 and BCA 256/010.

### Setting

The study was conducted in the DOV at Inkosi Albert Luthuli Central Hospital in KwaZulu-Natal province, South Africa. The DOV is an ISO 15189:2007 accredited laboratory within an academic quaternary-level hospital. The DOV does early infant diagnostic testing of DBS using HIV-1 PCR on the CAP/CTM analyser for all HIV-exposed infants in public health facilities in KwaZulu-Natal province. During the study period from January 2014 to December 2014, the South African antiretroviral treatment guidelines^11^ recommended that all HIV-exposed infants have an HIV-1 PCR test at age six weeks, at six weeks post-cessation of breastfeeding and at any age less than 18 months that the child may be symptomatic (after age 18 months, serology was used for HIV diagnosis). Paediatric guidelines recommend testing infants at the six-week immunisation visit and, for those who test positive, referral for confirmation of HIV infection and initiation of ART. Infants testing negative should be followed up and retested at age 18 months and/or after cessation of breastfeeding.^11,12^

### Laboratory database search

We searched our laboratory database for reports on routine HIV-1 PCR test requests that were rejected during 2014. The database is managed with TrakCare Lab software (Intersystems Corporation, Cambridge, Massachusetts, United States) and the data were downloaded and stored in Microsoft Excel 2010 files (Microsoft Corporation, Redmond, Washington, United States). The data extracted included specimen numbers, reasons for specimen rejections and patient demographics, including the health facility where the DBS was collected (each facility was assigned a two-letter code). We also extracted the results for all HIV-1 CAP/CTM PCR routine diagnostic tests conducted during 2014.

### Laboratory experiment to validate the two-spot method

#### Specimen preparation

**FIGURE 1:**
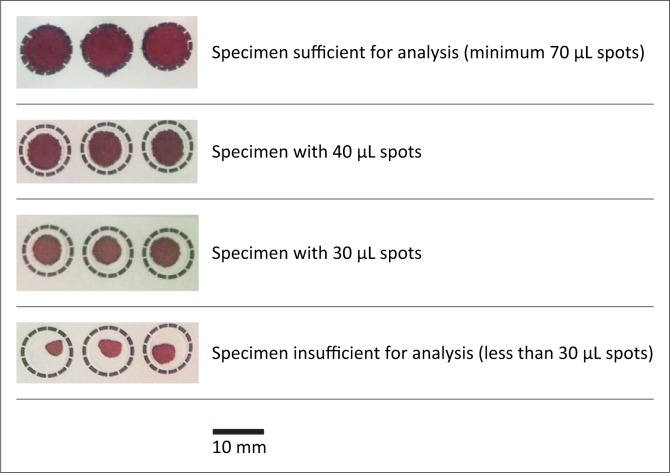
Example filter paper card with sufficient and insufficient blood spots.

Residual EDTA blood specimens from previous successful, routine diagnostic HIV-1 PCR tests were selected and anonymised using a number allocated for this experiment. Specimens with insufficient volume and specimens that were collected more than one day prior to testing were excluded. Blood samples from 20 HIV-negative and 20 HIV-positive specimens were selected for the validation study.^13^ Blood was pipetted onto pre-labelled SS906 filter paper cards by filling one circle completely (so that it contained 70 µL of blood), then filling two circles incompletely (so that one contained 30 µL and the other 40 µL of blood). Examples of sufficient and insufficient specimens are shown in Figure 1. The original specimen tubes were discarded as per routine biohazard waste disposal procedures. The blood spots were allowed to air-dry at room temperature. The sizes of the DBS were measured using calipers and recorded, then manually cut for HIV-1 PCR testing.

#### Validation experiment

The complete (70 µL) DBS were processed using the standard (one-spot) method: each blood spot was inserted into a specimen preparation tube with lysis buffer, which had 1000 µL of specimen pre-extraction reagent (30 mM sodium citrate dehydrate, 42.5% guanidine thiocyanate, 4% polydocanol and 2% dithiothreitol) added to each tube. The tube was then subjected to thermomixing in a Thermomixer comfort (Eppendorf, Hamburg, Germany) at 56 °C for 10 minutes at 1000 rpm in order to elute the DBS. The two incomplete (30 µL and 40 µL) DBS were then combined in a single specimen preparation tube and processed using the above method. For both sample types, the DBS were pushed to one side when inserted into the specimen preparation tube, as per the standard procedure.

Both types of DBS were then tested in parallel over a period of five days for HIV-1 nucleic acids using the CAP/CTM as per manufacturer’s instructions. Briefly, the specimen preparation tubes were transferred to specimen racks, then loaded into the CAP/CTM analyser, which is an automated closed system with combined extraction, real-time PCR amplification and detection allowing for reduced contamination. Known low-positive and negative controls were included with every batch. The analyser software automatically determined the validity of the run, then determined the presence of HIV-1 nucleic acids based on the Ct value, namely, the PCR cycle at which the signal indicated amplification.

An HIV standard (World Health Organization 3rd HIV-1 International Standard National Institute for Biological Standards and Control code: 10/152) was used to perform a dilution series and establish a lower limit of detection for the HIV-1 PCR test when using the two-spot method. Replicates of the HIV standard were tested to establish precision.^13^

### Statistical methods

Statistical analyses were performed using IBM SPSS Statistics for Windows, version 22.0 (IBM Corp., Armonk, New York, United States). For the analysis of the laboratory database search, the frequency of each reason for specimen rejection was determined and for each health facility, a percentage of tests rejected as a result of pre-analytical problems was determined out of the total number of HIV-1 PCR tests requested. The HIV-1 PCR results of specimens processed pre-analytically using the standard method were then compared with specimens processed using the two-spot method. The Chi-squared test was used to compare categorical variables. A *p*-value of < 0.05 was regarded as statistically significant.

For the analysis of the laboratory experiment to validate the two-spot method, measures of sensitivity and specificity were used to describe the accuracy of the method. A scatter plot of Ct values was used to compare the two methods. Precision was determined by calculating the standard deviation and coefficient of variance of replicates. A probit regression analysis was used to determine the lower limit of detection of the assay.

## Results

### Laboratory database search

The analysis of database records for 2014 revealed that 88 481 specimens were sent to DOV for HIV-1 PCR testing, of which 3.8% were rejected or had no results. Ninety-one (0.1%) test requests had no results as a result of analytical problems and 3276 (3.7%) were rejected because of pre-analytical problems (Table 1).

**TABLE 1:** Dried blood spot specimens received in 2014 at the Department of Virology laboratory at Inkosi Albert Luthuli Central Hospital, KwaZulu-Natal province, South Africa.

Outcome	Number of specimens (percentage of total)
All	88 481
Tested	85 114 (96.2%)
Rejected: pre-analytical problems	3276 (3.7%)
No results: analytical problems	91 (0.1%)

Insufficient specimen volume comprised 48.9% of the total number of rejections resulting from pre-analytical problems (Table 2). Other reasons for rejections included: inadequate information supplied on the test request form; incorrect clinical indication for the test; and poor specimen quality, such as incorrect specimen type, specimens that were too old for analysis and specimen cards that were expired.

**TABLE 2:** Dried blood spots rejected because of pre-analytical problems in 2014 at the Department of Virology laboratory at Inkosi Albert Luthuli Central Hospital, KwaZulu-Natal province, South Africa.

Reason for rejection	Number of specimens (percentage of total)
All	3276
Insufficient volume	1602 (48.9%)
Inadequate information	794 (24.2%)
Incorrect clinical indication	504 (15.4%)
Poor specimen quality	376 (11.5%)

**FIGURE 2:**
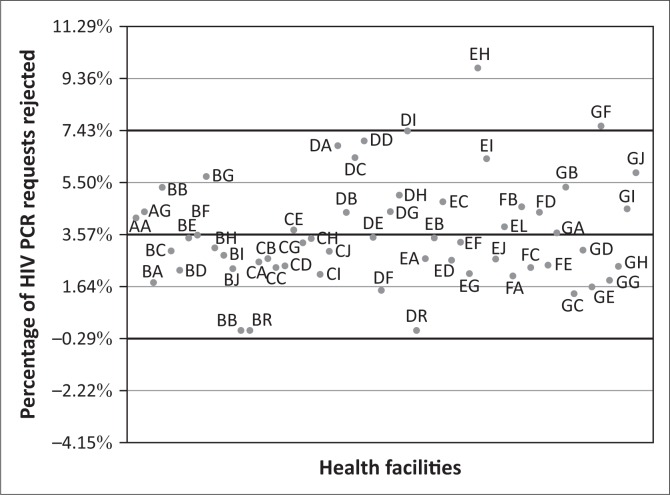
Percentage of HIV-1 PCR requests rejected because of pre-analytical factors by health facilities in KwaZulu-Natal province, South Africa, in 2014. Each health facility is represented by a two-letter code. The analysis included data from 88 481 dried blood spot specimens. The horizontal line at 3.57% indicates the mean percentage of rejected requests for all facilities. The horizontal lines at 7.43% and -0.29% indicate two standard deviations from the mean.

The data on the percentage of requests that were rejected per health facility were normally distributed (Figure 2). The mean of the percentages of tests rejected for all health facilities in KwaZulu-Natal was 3.6%. Two facilities (EH and GF, names blinded for publication purposes) were more than two standard deviations away from the mean, with rejection rates of 9.7% for EH and 7.6% for GF.

For all HIV-1 PCR test results in 2014 using the CAP/CTM assay, there was an overall indeterminate rate of 1.7% (Table 3). There were 10 307 valid positive and negative test results using the two-spot method. The two-spot method had an indeterminate rate of 1.9%.

**TABLE 3:** Results of specimens tested by standard method and two-spot method in 2014 at the Department of Virology laboratory at Inkosi Albert Luthuli Central Hospital, KwaZulu-Natal province, South Africa.

Result	All specimens tested	Specimens tested by the standard, one-spot method	Specimens tested by the two-spot method	Standard vs two-spot method *p*-value†
All	85 114	74 610	10 504	-
Positive	2967 (3.5%)	2651 (3.5%)	316 (3.0%)	0.0040
Negative	80 676 (94.8%)	70 685 (94.7%)	9991 (95.1%)	0.7961
Indeterminate	1471 (1.7%)	1274 (1.7%)	197 (1.9%)	0.2402

† *p*-values < 0.05 were considered statistically significant.

### Validation of the two-spot method

**FIGURE 3:**
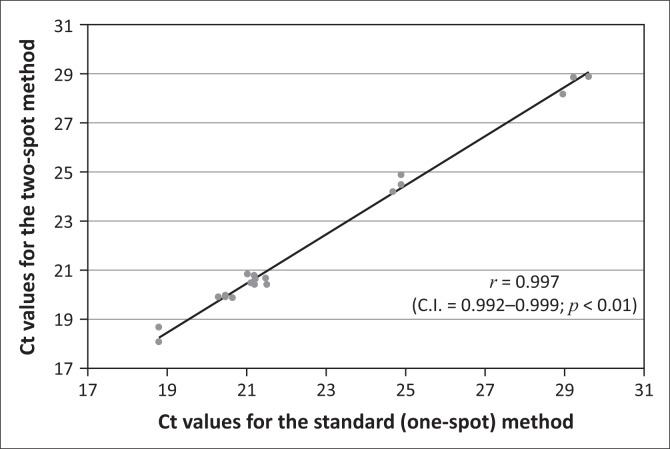
Validation experiment correlations between Ct values for the standard (one-spot) and two-spot specimen processing methods. Twenty positive and 20 negative specimens were processed by both the two-spot and the standard one-spot methods of pre-analytical specimen processing. The scatterplot shows the Ct values obtained during real-time PCR amplification of the positive specimens. An overlaid line of linearity and Pearson’s correlation coefficient of 0.997 shows the correlation between the Ct values for each of the specimen processing methods.

The average diameter of the 30 µL DBS was 8.99 mm (95% confidence interval: 8.89–9.12 mm) and for the 40 µL DBS was 10.08 mm (95% confidence interval: 9.95–10.21 mm). A comparison of the qualitative results of patient specimens showed 100% agreement in accuracy between the two methods. The comparison of the Ct values for the standard method versus the two-spot method showed that the Pearson correlation coefficient was 0.997 (Figure 3). In the precision study, 19 replicates of a specimen with a known concentration yielded a coefficient of variance of 0.0078. The probit regression analysis of the dilution series showed that 95% of replicates were detected at 3.6 log IU/mL (95% confidence interval: 2.9–4.925 log IU/mL).

## Discussion

This study is the first to describe the reasons for laboratory rejections of DBS specimens being submitted for HIV-1 PCR testing in KwaZulu-Natal province. Almost 4% of DBS specimens that were submitted to the laboratory were rejected because of pre-analytical problems. Our analysis showed that 48.9% of pre-analytical problems resulted from insufficient specimen volume and we identified two health facilities with high specimen rejection rates.

Insufficient DBS volume has been shown to affect the yield of HIV-1 nucleic acids^14^ and these specimens are therefore rejected by laboratories. Others have also described challenges in the field with specimen collection and processing, as a result of specimen quality factors such as mislabeling or insufficient volume. Rates of specimen rejection vary considerably and there is a paucity of data describing the frequency of each specimen quality issue.^15,16^ Data such as specimen quality should be analysed in order to review the performance of HIV-1 PCR testing in the field and to standardise DBS specimen collection methods, storage and testing.^14^ This study has shown that analysing specimen quality can identify health facilities which require targeted interventions, such as retraining of healthcare workers.

The Prevention of Mother-to-Child Transmission program in KwaZulu-Natal has shown significant success, largely because of the implementation of testing pregnant women and starting them on ART,^7^ but problems have been described in the follow-up of mothers and infants.^8,17^ It has been shown that delaying HIV diagnosis in HIV-exposed infants results in a failure to link infants to HIV care, significant loss to follow up and mortality.^18^ At immunisation clinics in KwaZulu-Natal, nurses perform the specimen collection, whereas in hospital wards and clinics, some specimens are submitted by attending doctors. It has been shown that intense training and mentoring of nurses significantly improves the uptake of infant HIV testing and the overall quality of care.^19^ However, this needs to be expanded and implemented on a much larger scale.^20,21^ The need to expand nurse training will increase with the planned implementation of HIV-1 DNA PCR testing of exposed neonates at birth.^22^ As this is being newly implemented, healthcare workers in labour wards and nurseries may not be as experienced with DBS specimen collection as those in immunisation clinics.

We have described an innovative two-spot method for pre-analytical specimen processing that can reduce the number of DBS specimen rejections resulting from insufficient specimen volume. This method has been implemented in DOV for approximately three years and is used as an adjunct to ongoing telephonic and electronic communication and training of nurses in the public healthcare sector. Personal communications with other laboratories indicate that the two-spot method is being used in other HIV-1 PCR testing laboratories in South Africa. However, to our knowledge the two-spot method has not yet been described in the scientific literature.

The specimens processed using the two-spot method in 2014 yielded over 10 000 additional results from specimens that would otherwise have been rejected. Furthermore, the proportion of specimens processed using the two-spot method with indeterminate results was comparable with that of all specimens processed in 2014.

The validation study found that the two-spot method correlated well with the standard method and had good precision. The lower limit of detection of the HIV-1 PCR test when using the two-spot method was 3.6 log IU/mL, which is comparable to the 2.7 log IU/mL stated on the package insert for the standard method. Future studies comparing the detection limits between the two methods may be required.

## Limitations

A major limitation with respect to analytical sensitivity is that the test is being used in infants within a Prevention of Mother to Child Transmission programme, most of whom have been exposed to ART. The presence of these drugs has been stated as a limitation by the manufacturer on the HIV-1 PCR package insert. Therefore, field evaluation of detection limits of the test may be required in a significantly ART-exposed patient population such as South Africa.^23^ Another limitation of our study is that the volumes of 30 µL and 40 µL used in the validation study were selected in an attempt to simulate the real-world situation. However, a range of volumes could be present in DBS specimens in the field, for example, 20 µL and 50 µL. A third limitation is that the proportion of positive results using the two-spot method was significantly lower compared with the standard one-spot method. We speculate that this difference may be because of inconsistent selection of specimens for two-spot processing. Practical application of the two-spot method would require an objective assessment of the DBS specimen to reduce the subjectivity of specimen processing in the laboratory. Mean circle diameters for 30 µL and 40 µL spots can be provided to laboratory assistants on a template in order to demonstrate acceptable specimen volumes and aid the selection of patient specimens which are suitable for the two-spot method. We are in the process of implementing the use of such a template based on the findings of this study. A follow-up database analysis will be required to assess whether this intervention is effective. Laboratories that plan to use or are currently using the two-spot method should consider this cautionary measure.

### Conclusion

In conclusion, we describe real-world problems with DBS specimen quality, which may have major implications for the management of HIV-infected infants in a resource-limited setting. The laboratory database can be used to monitor specimen quality and identify specific health facilities for interventions such as retraining of healthcare workers. As an adjunct to training, the two-spot method of specimen processing may be used to help reduce the number of specimens rejected. This method can potentially be applied to other HIV-exposed infant populations in resource-limited settings to reduce the need for patients having to repeat specimen collection and improve linkage to care.
